# Effect of Land Use History and Pattern on Soil Carbon Storage in Arid Region of Central Asia

**DOI:** 10.1371/journal.pone.0068372

**Published:** 2013-07-10

**Authors:** Xiaoyu Li, Yugang Wang, Lijuan Liu, Geping Luo, Yan Li, Xi Chen

**Affiliations:** State Key Lab of Desert and Oasis Ecology, Xinjiang Institute of Ecology and Geography, Chinese Academy of Sciences, Urumqi, Xinjiang, China; Roehampton university, United Kingdom

## Abstract

The purpose of this study is to investigate variations in soil organic carbon (SOC) in arid areas due to differences in the cultivation history, land use, and soil salinization. The study area is the lower Sangong River basin on the piedmont of the northern TianShan mountains, which experiences heavy land-use activities. In 1982 and 2005,127(152) and 74 (161) samples in old (new) oasis were collected from each site at the surface soil (i.e., 0–20 cm). The data reveal that the mean value of the surface soil organic carbon content of the old oasis was higher than that of the new oasis by 4.01 g/kg in 1982 and 3.79 g/kg in 2005. Additionally, the soil organic carbon content decreased more rapidly in the newly reclaimed oasis than in the old oasis from 1982 to 2005. The spatial pattern of the SOC content was correlated with the exploitation time in the new oasis, the agricultural land use history, and the SOC content. The decreasing trend is clearer in the high SOC content area than in the low SOC content area. Farmland is the largest carbon pool in both the new and old oases. The carbon density of the old oasis was higher than that of the new oasis by 4.01 and 3.79 g/kg in 1982 and 2005 respectively. The loss of SOC in the agricultural watershed of the arid region in NW China is obvious. Improvements of land management practices, such as no tillage, straw returning to soil, and balanced fertilization techniques, should be adopted to increase the SOC content.

## Introduction

Changes in land use and land cover are among the main human activities affecting the surface of the earth [1], with the expansion of agriculture posited as one of the main causes of land cover change globally. The role of terrestrial ecosystems as sources and sinks of C has been highlighted, underscoring the impact of land use and land cover changes on the global climate [2]–[5]. Globally, the soil organic carbon (SOC) stock is 1550 Pg (1 Pg = 10^15^ g) at a1-m depth [6]; it is as much as three times greater than the atmospheric carbon pool and double that in the biota [7]–[8]. Soils are the largest organic carbon sink on Earth. The abundance of organic C in the soil affects and is affected by land use and land cover changes, and organic carbon’s role as a key determinant of soil fertility and vegetation production has been documented in recent years [9]–[13]. The effects of land use change on the carbon cycle are complex and are likely to vary among land use types on different landscape scales [14]. Understanding the carbon cycle across different landscape scales, including patch, field, watershed, and global scales, is important [15]–[17]. Therefore, understanding the changes in the organic carbon storage space distribution in soil is crucial for assessing current regional, continental, and global soil C stores and predicting and ameliorating the consequences of global change [18].

The magnitude of the change in C storage depends on how physical, chemical, or biological processes are altered over time under different land uses [19]. The extent to which soils and vegetation act as CO_2_ sinks or sources depends largely on land-use management [20]. Appropriate land use management practices can improve SOC, whereas unreasonable practices reduce SOC [21]. Some of the land use activities that affect carbon fluxes in soils are deforestation, afforestation, biomass burning, cultivation, crop residue management, and the application of inorganic fertilizers and organic manure [20]. Generally, soil organic carbon (SOC) stocks under cropland are lower than those under pasture or forest, and forest SOC stocks tend to be higher than pasture SOC stocks. The conversion of forest to pasture or to cropland or the conversion of pasture to cropland decreases SOC stocks, whereas the opposite conversions usually lead to increased SOC stocks [22]–[24]. Historically, when croplands have been established on land previously used for native vegetation, the soil C pool has been a major source of atmospheric CO_2_, contributing approximately 180–200 Pg C over the last two centuries [25], which is approximately 40% of the total anthropogenic CO_2_ emissions [26]–[27]. At a global scale, land use and land cover changes (LULCC) are estimated to contribute 25% of the anthropogenic flux of carbon dioxide to the atmosphere, the second highest contribution after that of fossil fuels [28]. The amount of C released to the atmosphere after LULCC in the 1980s and 1990s is estimated as 1.41 and 1.6PgCyr^−1^ [29]; however, reducing CO_2_ emissions and increasing C sequestration by vegetation and soils can contribute to decreasing this rate [5], [30]–[31]. Davidson and Ackerman [32] suggested that nearly all C lost from soil occurs within 20 years and that most occurs within 5 years, after initial cultivation. Therefore, decreasing the atmospheric CO_2_ contraction is necessary, which requires understanding the variations in regional SOC stocks by land use history and the resulting patterns.

Arid and semi-arid lands cover approximately 45% of the global terrestrial area and contain 16% of the global soil carbon pool [33]. Overgrazing, human activities, and climate variation have contributed to the desertification and degradation of more than two-thirds of these fragile ecosystems [34]. Changes in the vegetation and the release of carbon from these drier regions must be considered when discussing carbon balance on a global scale [35]–[36]. Desertification is estimated to affect approximately 1.137 Bha of soils and an additional 2.576 Bha of rangeland vegetation in drylands around the world. Furthermore, the total historic loss of C due to desertification may be 18–28 Pg [37], indicating that land desertification characterized by soil degradation and the diminution or destruction of the biological potential of ecosystems have played an important role in the atmospheric CO_2_enrichment [38].

China is among the countries seriously affected by desertification. It is estimated that dryland and susceptible dryland (excluding hyperarid areas) comprise357.05 and 331.70 million ha of China, respectively, and in 2004, 263.62 million ha had suffered or was suffering from desertification [39]. Feng et al. [40] estimated that desert lands in north China caused a net emission of 2.17 Pg of C from the 1950s to the 1990s.

Xinjiang, the Uighur Autonomous Region, lies in the hinterland of the Eurasian continent, far from the ocean, in what was known in China as the Western Territory in ancient times. Its 1.66 million sq km represents approximately one-sixth of the total territory of China, making it the largest of China's regions and provinces. Surrounded by mountains, Xinjiang features a temperate continental climate with low precipitation. It has semi-arid and arid climates, with a long-term average annual precipitation of 130 mm and a long-term average of 55 rainy days per year. It is characterized by widespread desert and a fragile ecosystem. Over the past 50 years, the local vegetation and soil have been changed or modified by large-scale land reclamation, irrigation, and cultivation, as well as the application of fertilizers [41]. The farmland area and population increased by 63.5% and 80.1% during 1949–2011 [42]. In particular, intensive soil cultivation was initiated in the1950s. An overuse of water resources, expansion of agricultural lands, and environmental degradation have led to severe problems, such as soil organic carbon loss in Xinjiang oases [43]. The knowledge of the spatial variation of SOC and itsrelation to land use and soil properties is thus crucial for enacting more rational management measures at regional levels [44].

**Figure 1:**
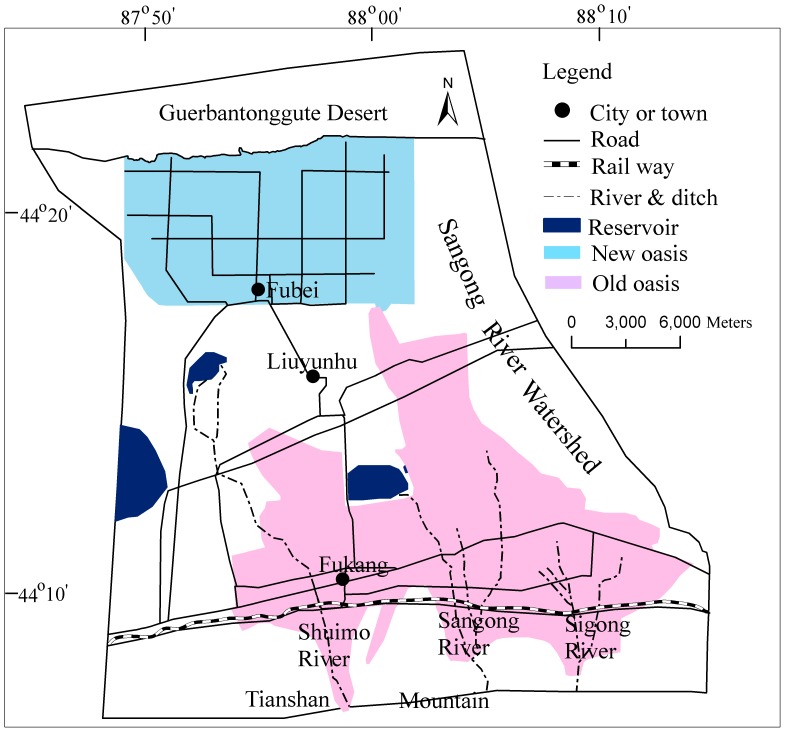
Study area of the Sangong river basin and its oases.

Thus, the objective of this work was to estimate SOC in arid areas as a function of cultivation history, land use, and soil salinization. The study site was the lower Sangong River basin on the piedmont of the northern TianShan mountains, which features heavy land-use activities (Fig. 1). The goal was to identify land use and soil management options to help achieve the full SOC sequestration potential.

## Materials and Methods

### Study Area

For the Sangong river cachment, where the 308 surface soil samples (0–20 cm) were collected in October 2005, no specific permissions were required for these locations. Thus the soil sampling in the agricultural region was permitted and no specific permissions were required.

We confirmed that the field studies did not involve any endangered or protected species.

The Sangong River basin, a typical inland river basin with spring and autumn flooding, is located in the northernTianShan mountains and the southern Guerbantonggute Desert in central Asia, extending between 87°47′-88°17′ E and 44°09′-44°29′ N (Fig. 1). The Sangong River originates from the northern slope of the TianShan mountains. The elevation along the basin descends from 750 m (the southern region) to 450 m (the northern region) above sea level. Passing by plain oases in the middle, it reaches the southern edge of the Gurbantunggut Desert, a total distance of less than 80 km. The basin is a mountain-oasis-desert landscape pattern typical of the region. Oases, more productive than the surrounding deserts, are the primary sites for human settlement because of the availability of fertile soil, fresh groundwater and surface runoff from the nearby mountains [41].

The southern part of the oasis region is an old oasis with thousands of years of agricultural history; however, the northern part has featured agricultural production for fewer than 60 years and is thus a new oasis. The boundary of new oasis is straight-sided because of this area was reclaimed from desert land by XinJiang Production and Construction Group in 1950s under unified planning. And the boundary of old oasis is curled naturally because of this area was developed from natural land for hundreds years. A reservoir was constructed in the 1970s at the end of the Sangong River to supply water for agricultural irrigation. These hydrological and land-use changes have promoted soil salinization and land degradation in the entire lower reaches of the Sangong River catchment [45].

The climate is an arid continental climate with an annual precipitation of 163 mm and an annual pan evaporation of 1780–2460 mm. The main soil type is Solonchak, covering approximately 37% of the study area; less frequent soil types include Haplic calcisols and Aquert on substrates of varied fertility. The natural vegetation of the region is characterized by different types of xeric or halophyte communities, dominated by the desert shrubs *Tamarix ramosissima* and *Haloxylon ammodendron* as well as *Reaumuria soongorica* scrubs. Crops in the oasis include cotton, wheat, hops, grapes, and corn.

### Soil Sampling and Measurements

In 1982, soil samples were collected in the Sangong river watershed as part of China’s National Soil Inventory of the 1980s [46]. In all, 127 surface soil (0–20 cm) samples from the old oasis and 152 samples from the new oasis were positioned according to the recorded geographic location and land cover information. The soil organic matter for each soil sample was converted to soil organic carbon (SOC) by multiplying by 0.58 [47]–[49].

In 2005, a total of 308 surface soil samples (0–20 cm) were collected in different landscape types throughout the catchment oasis in October. Among these samples, 74 samples were obtained from the old oasis and 161 samples represented the new oasis. The sample sites covered various land use types. Soil samples from these sites were obtained by hand soil auger and analyzed in the laboratory. Samples were air-dried and crushed to pass through a 2-mm mesh. All of the soil samples were analyzed for soil salt content and soil organic matter, and the soil organic matter were converted to soil organic carbon (SOC) by multiplying by 0.58. Similarly to those in 1982.

An equation was developed to quantify and characterize the soil organic carbon storage state in different land-use types based on the distribution of soil organic carbon content by land-use type. For an individual land-use type with k patches, the equations below were used to calculate the soil organic carbon storage (g):
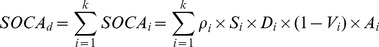
(1)

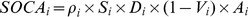
(2)where *k* is the number of patches, *ρ_i_* is the bulk density (Mg m^–3^), *S_i_* is the distribution area of soil organic carbon content (m^2^) for a degree of soil organic content *i*, *D_i_* is the thickness of soil layer (m), *V_i_* is the volume fraction of fragments >2 mm, and *A_i_* is the mean of the soil organic content (g kg^–1^). The 1982 soil bulk density data [46] and the 2005 soil bulk density under the same conditions and land-use type were determined using a soil corer (100-cm^3^stainless steel cylinder).

### Land Use Data

**Figure 2:**
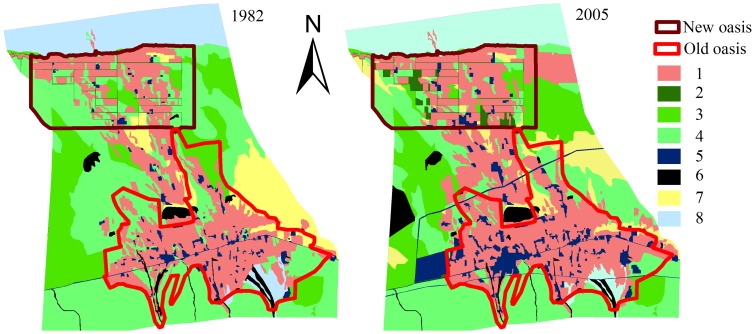
Land use map of the Sangong river basin and its oases in 1982 and 2005 (1, Farmland; 2, planted forest; 3, shrub land; 4, grassland; 5, construction land; 6, reservoir/flood land; 7, saline alkali land; 8, bald land).

The 1982 and 2005 land use data were obtained from remote sensing images, topography maps, and field investigations. A topographic map at the scale of 1∶50,000 was used to create a 1∶50,000image. In 2004, spot imagery (June, with 10×10 m resolution) was geo-referenced to the Universal Transverse Mercator projection with a root mean square (RMS) error of 0.45 pixels. To obtain the map of land uses in 2005, the land use types were investigated in June 2005 according to the maps of spot imagery, topography, and field. Using the rectified 2004 image as a basis, the 1982 image was rectified with an RMS error of 0.5 pixels (15 m). Visual interpretations of Landsat imagery have been demonstrated to be useful for land cover and vegetation mapping [50]. Land use types in the study area were divided into eight types: farmland, planted forest, shrub, grassland, construction land, reservoir/flood land, saline alkali land, and bald land (Fig. 2).

## Results and Discussion

### Land Use Change

As shown in Fig. 2 and Table 1, farmland is the main type of land use in both the new and old oases of the Sangong River watershed, accounting for 42.37% of the new oasis in 1982 and 50.49% in 2005 and accounting for 50.22% of the old oasis in 1982 and 58.56% in 2005. The farmland area increased by 8% from 1982 to 2005 in both the new and old oases. At the same time, grassland, the second largest land use type, decreased by 11.85% in the new oasis and 9.76% in the old oasis. Much of the grassland was converted into farmland over the last 25 years. The contribution of planted forest increased by 6.22% in the new oasis and was almost constant in the old oasis because land exploitation in the new oasis has been accompanied by the planting of shelter forest since 2000 due to land integrated management (Wang & Li, 2013). The shrub land increased by 3.98% in the new oasis and decreased by 5.03% in the old oasis. The flood land in the new oasis was disappeared in 2005 because of the runoff was dammed to the reservoir, which located at the upper stream of the new oasis, and the flood land was reclaimed to extend the farmland. In both the new and old oases, the landscape patch changed dramatically from 1982 to 2005 (Fig. 2). These remarkable transformations were responsible for the intensification of human activities in the oases. Socioeconomic factors created by human activities are the main driver of land-use change in shaping the agricultural land-use pattern [51]–[53].

**Table 1:** Area and percentage of land use types in the new and old oases in 1982 and 2005.

	New oasis				Old oasis			
Land use type	1982		2005		1982		2005	
	Area(hm)	%	Area(hm)	%	Area(hm)	%	Area(hm)	%
Farmland	6313.42	42.37%	7523.07	50.49%	12058.27	50.22%	14061.12	58.56%
Planted forest	465.55	3.12%	1391.07	9.34%	2.94	0.01%	9.18	0.04%
Shrub land	1298.33	8.71%	1891.07	12.69%	1462.87	6.09%	255.36	1.06%
Grassland	6090.79	40.88%	2835.01	19.03%	6025.54	25.10%	3682.24	15.34%
Construction land	278.24	1.87%	548.78	3.68%	1465.14	6.10%	3363.02	14.01%
Reservoir/flood land	29.02	0.19%	0	0.00%	869.31	3.62%	667.24	2.78%
Saline alkali land	411.16	2.76%	677.52	4.55%	1176.24	4.90%	1150.26	4.79%
Bald land	13.33	0.09%	33.06	0.22%	950.46	3.96%	822.24	3.42%
Total	14900	100%	14900	100%	24010.78	100%	24010.68	100%

### Descriptive Characteristics of Soil Organic Carbon

In the old oasis, the average surface soil organic carbon (SOC) was 11.44 g/kg in 1982, with a range of 4.93 to 19.89 g/kg, and 9.812 g/kg in 2005, with a range of 1.33 to 18.30 g/kg (Table 2). The average SOC content decreased by 14.2% from 1982 to 2005.

**Table 2:** Soil organic carbon (SOC) content (g/kg) in the Sangong river basin and its oases in 1982 and 2005.

	Minimum	Maximum	Mean	Median	SD	Skewness	Kurtosis	
Whole basin 1982	1.91	19.89	9.25	8.70	3.87	0.23	−0.87	
Whole basin 2005	0.58	21.71	7.15	6.55	3.64	0.66	0.32	
New oasis 1982	1.91	16.00	7.43	6.61	3.39	0.70	−0.35	
New oasis 2005	.58	14.82	6.03	5.39	2.92	0.76	−0.02	
Old oasis 1982	4.93	19.89	11.44	11.60	3.23	0.01	−0.60	
Old oasis 2005	1.33	18.30	9.81	10.21	3.25	0.01	0.52	

In the new oasis, the average SOC content was 7.428 g/kg in 1982, with a range of 1.91 to 16.00 g/kg, and 6.026 g/kg in 2005, with a range of 0.58 to 14.82 g/kg (Table 2). The average SOC content decreased by 18.9% from 1982 to 2005.

The mean SOC content in the old oasis is higher than that of the new oasis by 4.01 g/kg in 1982 and 3.79 g/kg in 2005. This difference may be the attributed to the fact that the old oasis was cultivated for over 500 years and is located in the middle river of the watershed, with good soil and geographical conditions. However, in both the old and new oases, the surface SOC decreased over the past 25years. The decrease is more rapid for the newly reclaimed oasis than the old oasis, indicating that the SOC content decrease improved by human activity is stronger in the surface soil, especially in the new oasis region. The result of land reclamation converted natural ecosystems to agricultural ecosystems, which generally depletes the SOC pool because of the lower C input and higher decomposition rates of agricultural ecosystems [54]–[55]. Furthermore, the change in SOC content varies between the old and new oasis due to differences in soil moisture and temperature regimes caused by plowing, drainage, biomass burning, and residue removal [56]–[57].

### Spatial Pattern of Soil Organic Carbon (SOC)

**Figure 3:**
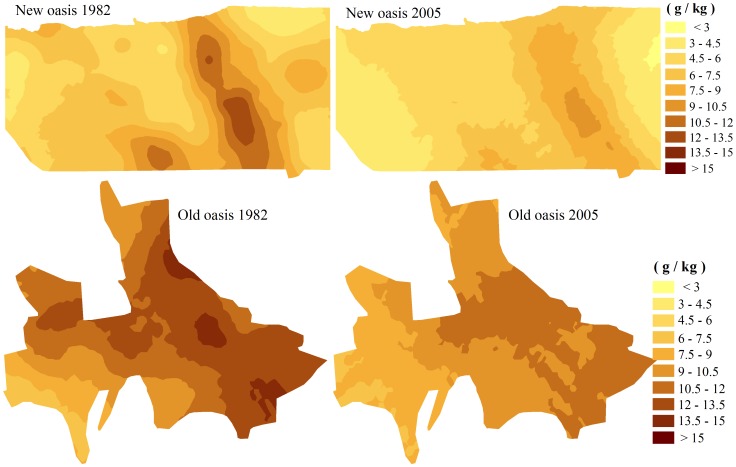
Estimated SOC content maps in the new and old oasis in 1982 and 2005 (mg/kg).

To describe the time-space distribution of SOC precisely and simply, spatial distribution maps of SOC in new and old oases in 1982 and 2005 were compiled by geostatistics (Table 3, Fig. 3). According to semivariogram calculation (Table 3), the experimental semivariogram of SOC content could be fitted with the exponential model for the old oasis in 1982, the spherical model for the old oasis in 2005, and the Gaussian model for new oasis in 1982 and 2005. The theoretical variation function and experimental variation function exhibits a better fitting (Table 3). The coefficients of determination (R^2^) were >0.55. The values of R were significant at the 0.01 level by F test, which shows that the semivariogram models well reflect the spatial structural characteristics of the SOC content. The ratio of nugget and sill (C0/C0+C) was between 0.25 and 0.75, except for in old oasis in 2005, suggesting moderate spatial autocorrelation [58]. However, the nugget to sill ratio for the SOC content in the old oasis in 2005 was <0.25, strong suggesting that the spatial dependence of SOC content was mainly due to structural factors in this case. The spatial variability of SOC content might be directly or indirectly caused by natural and human action on soil processes in the region, e.g., crop sequences, irrigation, precipitation, evaporation, and residue management [45].

**Table 3:** Parameters and F-test of fitted semivariogram models for soil organic carbon in oases.

	Theoretical model	Nugget (C_0_)	Sill (C_0_+C)	C/(C_0_+C)	Range	R^2^	RSS	F test
New oasis 1982	Gaussian model	5.21	12.49	0.583	4156.9	0.982	1.30	545.56**
New oasis 2005	Gaussian model	2.88	8.492	0.661	2303.6	0.862	4.15	62.46**
Old oasis 1982	Exponential model	5.50	12.45	0.558	19290	0.845	6.04	54.51**
Old oasis 2005	Spherical model	0.17	10.37	0.984	1550	0.547	10.50	12.07**

** Significance at α = 0.01 level of F test.

In the new oasis (Fig. 3), the high SOC content was distributed in the eastern part of the center region the SOC content generally decreased from the center region to the eastern and western regions. The SOC content was clearly correlated with the exploitation time. The exploitation of the new oasis began in the eastern part of the center region in the 1950s and then expanded westward. The shorter the agricultural land use history, the lower the SOC content. The SOC content decreased dramatically from 1982 to 2005. The decreasing trend is clearer in the high SOC content area than in the low SOC content area, indicating that land-use change in the form of land reclamation was accompanied by SOC content loss in surface soil between 1982 and 2005. This inference was also consistent with the conclusion that conversion of land-use types induces SOC content change [59].

The area of the new oasis with SOC contents above 10.5 g/kg (Table 4) decreased from 1434.34 ha (9.63%) in 1982, 76.15% of which was distributed in farmland, to 0 in 2005. The area with SOC contents of 7.5–10.5 g/kg decreased by 1357.54 ha (9.11%), 68.41% of which was from grassland. The area with SOC contents below 4.5 g/kg increased from 808.72 ha (5.43%) in 1982 to 3080.98 ha (20.68%) in 2005, a change of2272.26 ha (15.25%), of which 38.03% was shrub land and 36.36% was grassland. The area with an SOC content of 4.5–7.5 g/kg reached 8860.41 ha (59.47%) in 1982 and 9379.78 ha (62.95%) in 2005, of which the farmland area increased by 2067.49 ha and the grassland area decreased by 2921.75 ha from 1982 to 2005. Over the past 25 years, the SOC content in the new oasis decreased dramatically in the farmland, shrub land, and grassland.

**Table 4:** Area percent (%) of SOC content groups in different land use types in the new and old oases in 1982 and 2005.

			SOC content groups (g/kg)									
Land use type	Oasis	Period	<3	3–4.5	4.5–6	6–7.5	7.5–9	9–10.5	10.5–12	12–13.5	>13.5	Total
1	New	1982	–	2.38	10.93	9.32	6.39	6.02	5.53	1.80	–	42.37
		2005	0.78	3.47	21.37	12.76	10.22	1.88	–	–	–	50.49
	Old	1982	–	–	–	2.65	2.12	8.21	14.81	19.80	2.65	50.22
		2005	–	–	–	2.17	12.85	21.85	21.70	–	–	58.56
2	New	1982	–	0.04	1.03	0.91	0.38	0.47	0.20	0.10	–	3.12
		2005	–	0.66	5.18	1.83	1.47	0.21	–	–	–	9.34
	Old	1982	–	–	–	–	–	0.01	–	–	–	0.01
		2005	–	–	–	0.02	0.01	0.01	–	–	–	0.04
3	New	1982	–	–	3.12	3.37	2.00	0.22	–	–	–	8.71
		2005	0.03	5.77	5.31	1.52	0.06	–	–	–	–	12.69
	Old	1982	–	–	–	0.08	0.03	1.50	2.32	1.29	0.87	6.09
		2005	–	–	–	–	0.21	0.31	0.55	–	–	1.06
4	New	1982	–	2.45	11.44	17.89	5.06	2.50	1.38	0.18	–	40.88
		2005	–	7.99	6.57	3.15	1.14	0.18	–	–	–	19.03
	Old	1982	–	–	–	1.62	3.22	4.06	6.52	8.95	0.72	25.09
		2005	–	–	–	1.08	4.50	5.85	3.91	–	–	15.34
5	New	1982	–	–	0.36	0.32	0.38	0.47	0.21	0.12	–	1.87
		2005	–	–	1.08	1.67	0.90	0.03	–	–	–	3.68
	Old	1982	–	–	–	0.19	0.20	1.46	1.43	2.44	0.38	6.10
		2005	–	–	–	0.94	4.53	4.62	3.93	–	–	14.01
7	New	1982	–	–	0.01	0.11	0.07	–	–	–	–	0.19
		2005	–	–	–	–	–	–	–	–	–	–
	Old	1982	–	–	–	0.38	0.62	0.35	0.74	1.44	0.09	3.62
		2005	–	–	–	0.14	0.66	1.11	0.86	–	–	2.78
8	New	1982	–	0.54	0.58	0.02	0.81	0.70	0.11	–	–	2.76
		2005	–	1.97	1.18	1.13	0.28	–	–	–	–	4.55
	Old	1982	–	–	–	–	–	0.29	1.74	2.51	0.37	4.90
		2005	–	–	–	–	0.45	2.85	1.49	–	–	4.79
9	New	1982	–	0.03	0.05	0.01	–	–	–	–	–	0.09
		2005	–	–	0.17	0.06	–	–	–	–	–	0.22
	Old	1982	–	–	–	–	–	0.21	0.62	2.91	0.22	3.96
		2005	–	–	–	–	–	1.37	2.05	–	–	3.42

1, Farmland; 2, planted forest; 3, shrub land; 4, grassland; 5, construction land; 6, reservoir/flood land; 7, saline alkali land; 8, bald land.

In the old oasis (Fig. 3), the spatial distribution of SOC content in 1982 and 2005 shows that the SOC exhibited an obvious spatial distribution pattern, gradually decreasing from east to west. Temporally, the SOC content in the old oasis also decreased substantially from 1982 to 2005.

The area with an SOC content above 12 g/kg, which was mostly distributed in the eastern region of the oasis, accounted for 44.64% (10717.55 ha) of the total area in 1982, of which farmland and grassland occupied 50.28% and 21.66%, respectively. However, this area decreased to 0 in 2005. At the same time, the areas with SOC contents of 9–12 g/kg and below 9 g/kg increased from 44.26% (10627.37 ha) and 11.10% (2665.86 ha) in 1982 to 72.44% (17394.36 ha) and 27.56% (6616.32 ha) in 2005, respectively. Among the increases in low SOC content areas, the loss of SOC from the surface soil of farmland was most remarkable (68.98%).

### Effect of Land Use History and Land Use Type on Soil Organic Carbon (SOC)

**Figure 4:**
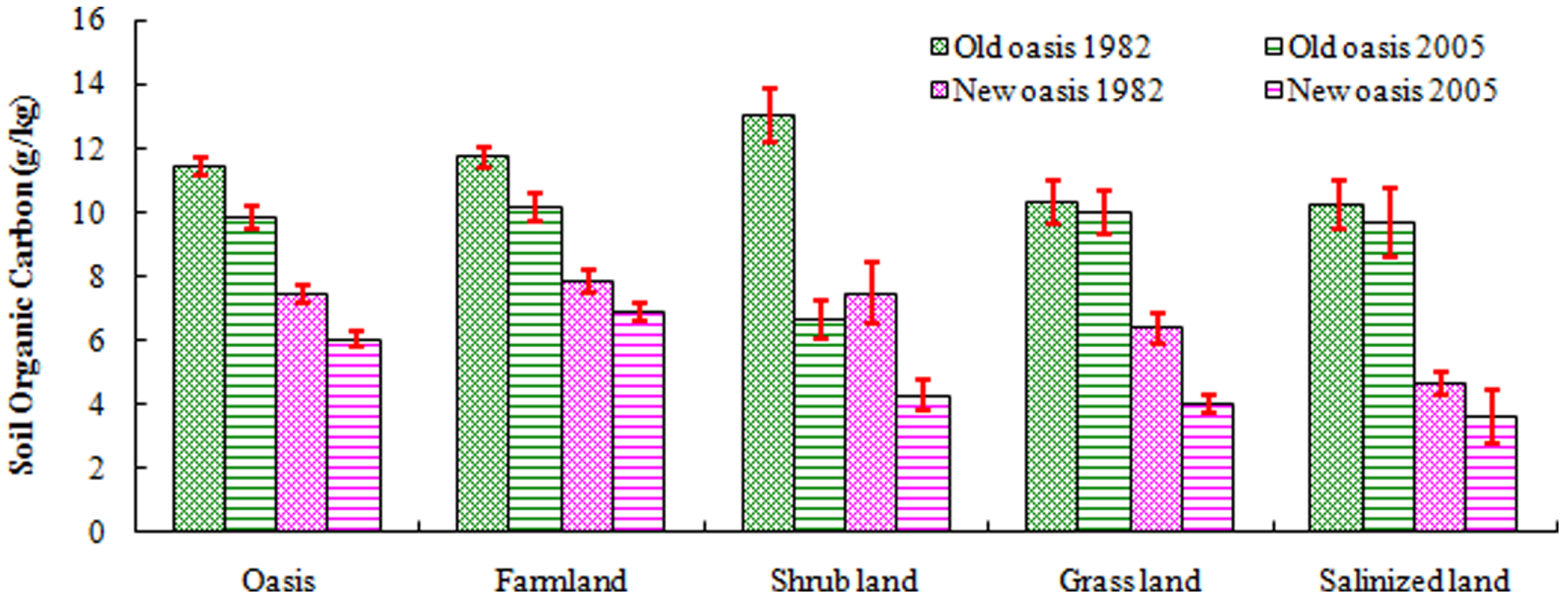
Mean SOC content and standard error in the oasis and main land use types.

Fig. 4 shows the SOC content by land use. The representative SOC content of the 4 main land use types in arid areas ranged from 4.640 g/kg in salinized land to12.998 g/kg in shrub land in 1982 and from 3.608 g/kg in salinized land to 10.125 g/kg in farmland in 2005.

In the old oasis, relative to the 1982 levels, the SOC decreased by 1.597 g/kg in farmland, 6.365 g/kg in shrub land, 0.331 g/kg in grassland, and 0.588 g/kg in salinized land in 2005, accounting for 13.62%, 48.97%, 3.21%, and 5.74% of the 1982 values, respectively. The SOC content of shrub land was higher than that of any other land use type in 1982 and decreased most rapidly over the last 20 years.

In both 1982 and 2005, the SOC of farmland was highest among the land use types in the new oasis. The decrease in SOC from 1982 to 2005 varied from 0.952 g/kg in farmland to 3.225 g/kg in shrub land. The contribution to the decrease in SOC was 12.19% for farmland, 43.18% for shrub land, 37.69% for grassland, and 22.23% for salinized land in 1982. Just as in the old oasis, the largest decrease occurred for shrub land. The SOC in grassland also decreased significantly.

As shown in Table 4, in the farmland in the new oasis, the area with SOC contents above 9 g/kg decreased from 13.35% of the new oasis area in 1982 to 1.88% in 2005, and the area with SOC contents below 9 g/kg increased from 29.02% in 1982 to 48.60% in 2005. In the farmland in the old oasis, the area with SOC contents above 12 g/kg decreased from 22.45% in 1982 to 0% in 2005, and the area with SOC contents below 12 g/kg increased from 27.79% in 1982 to 58.57% in 2005. This finding clearly shows the loss of SOC in farmland from 1982 to 2005 in both the new and old oasis. Grassland is the second most important land use type in the study area. The area of grassland in the new oasis with SOC content above 4.5 g/kg decreased from 38.45% of the new oasis area in 1982 to 11.04% in 2005, and the area with SOC contents below 4.5 g/kg increased from 2.445% in 1982 to 7.99% in 2005. In the old oasis, the grassland area with SOC contents above 10.5 g/kg decreased from 16.19% of the old oasis area in 1982 to 3.91% in 2005, and the area with SOC below 10.5 g/kg increased from 8.9% in 1982 to 11.43% in 2005.

### Dynamics of Land Use and Carbon Storage

In the new oasis, the total SOC storage was 306373.43 t (20.56 t/ha) in 1982 and 251234.78 t (16.86 t/ha) in 2005, a decrease of 55138.65 t (18.00%). Farmland is the largest carbon pool, accounting for 49.44% and 58.46% of the total SOC storage in 1982 and 2005, respectively. Although the farmland area increased by 1209.65 ha and 8.12% of the total new oasis area, the carbon storage in farmland decreased from 151469.56 t in 1982 to 146861.21 t in 2005, which is a decrease of4608.35 t or 3.04% of that in 1982, because of the decrease in the carbon density from23.99 t/ha to 19.52 t/ha. In grassland, the second largest carbon pool, the SOC storage decreased from 109317.91 t in 1982 to 38678.44 t in 2005, a decrease of70639.48 t or 64.62% of that in 1982. The grassland area and carbon density also decreased from 6090.79 ha (40.88%) and 17.95 t/ha in 1982 to 2835.01 ha (19.03%) and 13.64 t/ha in 2005, respectively. Shrub land also contributes substantially to SOC storage. With the shrub land area increased by 4% of the oasis area, the SOC storage increased by 895.92 t and the carbon density decreased from 15.66 t/ha to 11.22 t/ha.

In the old oasis, the total SOC storage and mean carbon density were 805616.15 t and 33.55 t/ha in 1982 and 722257.97 t and 30.08 t/ha in 2005, respectively. The carbon storage and density decreased by 10.35% over the past 25 years. The carbon density of the old oasis was much higher than that of the new oasis at the same time, reaching 1.63 and 1.78 times that of the new oasis in 1982 and 2005, respectively. Farmland is the dominant land use type, and 50.22% of the land area accounted for 53.26% of the SOC storage in 1982 and 58.56% of the land area accounted for 59.92% of the SOC storage in 2005. The carbon density of the farmland decreased from 35.58 t/ha in 1982 to 30.78 t/ha in 2005 (13.49%). Grassland is the second largest land use type in the old oasis; 25.10% of the land area accounted for 21.97% of the SOC storage in 1982, and 15.34% of the land area accounted for 12.95% of the SOC storage in 2005. The carbon density of grassland decreased from 29.37 t/ha in 1982 to 25.41 t/ha in 2005 (13.50%). Due to the decreases in the area and carbon density of grassland, the SOC storage in grassland decreased by 47.14%. However, the change of the carbon density value of the old oasis is not significantly different than that of new oasis between 1982 and 2005. This result indicated that the soil carbon storage ability is the same throughout the region, regardless of the age of the oasis, between 1982 and 2005. The loss of SOC in each land-use type was <8 Mg C/ha, and the amount of SOC lost varies by land-use type in the past 25 years (Table 5). The loss of SOC may reach20–50 Mg C/ha within the first 5–50 years after conversion [60]. In the topsoil, the carbon process in the oasis was mainly dominated by consumption and agricultural cultivation, which may produce unexpectedly high carbon stocks in deep soil but carbon loss in the topsoil [61]. Obviously, the dynamics of land use in the oasis can cause SOC loss and thereby reduce the carbon storage in the surface soil.

**Table 5:** SOC storage and density of different land use types in the new and old oases in 1982 and 2005.

			1	2	3	4	5	7	8	9	Total or Mean
	1982	Carbon storage (t)	151469.56	9345.29	20327.22	109317.91	8540.69	697.44	6479.70	195.63	306373.43
New		Carbon density(t/ha)	23.99	20.07	15.66	17.95	30.70	24.03	15.76	14.67	20.35
Oasis	2005	Carbon storage (t)	146861.21	22898.98	21223.14	38678.44	13390.58	0	7613.31	569.13	251234.78
		Carbon density(t/ha)	19.52	16.46	11.22	13.64	24.40		11.24	17.21	16.24
	1982	Carbon storage (t)	429035.88	78.57	39995.96	176975.59	61104.50	30986.22	31212.32	36227.11	805616.15
Old		Carbon density(t/ha)	35.58	26.72	27.34	29.37	41.71	35.64	26.54	38.12	32.63
Oasis	2005	Carbon storage (t)	432812.28	195.96	6164.93	93553.02	116109.36	21366.45	25263.57	26792.40	722257.97
		Carbon density(t/ha)	30.78	21.343	24.14	25.41	34.53	32.02	21.96	32.58	27.84

1, Farmland; 2, planted forest; 3, shrub land; 4, grassland; 5, construction land; 6, reservoir/flood land; 7, saline alkali land; 8, bald land.

### Effects of Soil Salinity on SOC Content

**Figure 5:**
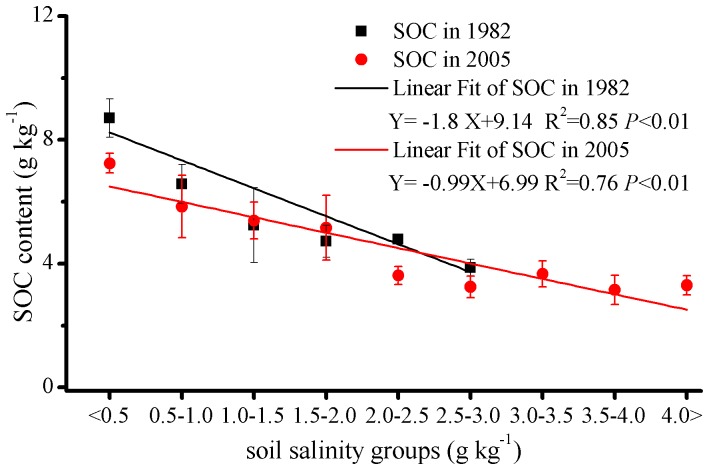
Relationship between SOC content and soil salinity.

In recent years, the study area has experienced severe soil salinization as a result of agricultural land exploitation. Agricultural land exploitation requires irrigation, which directly increases the groundwater table, and the resultant higher evaporation leads to soil salinization [45]. The correlations between SOC content and soil salinity were analyzed for the new oasis (Fig. 5). We grouped the samples (152 samples in 1982 and 161 samples in 2005) by soil salinity to make the relationship between SOC and soil salinity more clear (Fig. 5).

The SOC content was negatively correlated with soil salinity in both 1982 and 2005 (p<0.01), the analysis of covariance showed that the mean difference is significant at the 0.05 level. This suggests that soil salinization can affect SOC content. Because soil salinity decreases plant productivity and hence the input of C into the soil, it also affects microbial activity and therefore SOC decomposition rates [62]. The dramatic salt accumulation was a result of agricultural land exploitation, which requires the oasis to be irrigated. Water consumption by agricultural irrigates may leach SOC into the subsoil. The SOC is easily transported by irrigation water because of its relatively low density (<1.8 Mg/m^3^) [59] and is therefore concentrated near the soil surface. Thus, the SOC content was decreased in the artificial landscape of both the new and old oasis, including farmland, planted forest, etc. Therefore, much more effort is needed to decrease soil salinity and then increase SOC content by adjusting land-use structure as an effective strategy for both the new and old oases.

### Conclusion

Long-term loss of soil organic matter is a major threat to sustainable agriculture in China. This study investigated the SOC dynamics under different land use histories and patterns in a typical agricultural watershed in an arid area of NW China. The farmland area increased by 8% from 1982 to 2005 in both the new oasis and the old oasis, whereas grassland, the second largest land use type, decreased by 11.85% in the new oasis and 9.76% in the old oasis. Farmland expansion at the expense of shrub land and grassland is a common pattern in the arid region.

The mean surface soil organic carbon (SOC) was higher in the old oasis than in the new oasis by 4.01 g/kg in 1982 and 3.79 g/kg in 2005. Additionally, the SOC decrease in the newly reclaimed oasis is more rapid than that in the old oasis. Spatially, the SOC content pattern was clearly correlated with the exploitation time in the new oasis: the shorter the agricultural land use history, the lower the SOC content. The decreasing trend in the high SOC content area is clearer than that in the low SOC content area. In the new oasis, the total SOC storage was 306373.43 t (20.56 t/ha) in 1982 and 251234.78 t (16.86 t/ha) in 2005, a decrease of 55138.65 t (18.00%). Farmland is the largest carbon pool in the new oasis, with the highest SOC content, accounting for 49.44% and 58.46% of the total SOC storage in 1982 and 2005, respectively. In the old oasis, the total SOC storage was 805616.15 t in 1982 and 722257.97 t in 2005 and decreased by 10.35% over the past25 years. The carbon density of the old oasis was much higher than that of the new oasis at the same time, reaching 1.63 and 1.78 times that of the new oasis in 1982 and 2005, respectively. The loss of SOC in the agricultural watershed of the arid region in NW China is substantial, and the change in carbon density does not differ significantly between the old oasis and new oasis but does vary among land use types.

The loss of SOC in the agricultural watershed of the arid region in NW China is high. Best management practices are likely to stabilize and reduce carbon release from soils, as well as increase further the storage of carbon in terrestrial ecosystems [20], [63]. Land management practices should be improved by the implementation of such approaches as no tillage, straw returning to soil, and balanced fertilization techniques to increase the SOC content.
